# Novel recombinant SARS-CoV-2 lineage detected through genomic surveillance in Wales, UK

**DOI:** 10.1099/mgen.0.000984

**Published:** 2023-04-13

**Authors:** Nicole Pacchiarini, Michelle Cronin, Clare Sawyer, Catie Williams, Andrew Beazer, Simon Cottrell, Mari Morgan, Vince Saunders, Catherine Moore, Thomas R. Connor, Christopher Williams

**Affiliations:** ^1^​ Communicable Disease Surveillance Centre (CDSC), Public Health Wales, Cardiff, Wales, UK; ^2^​ Pathogen Genomics Unit, Public Health Wales, Cardiff, Wales, UK; ^3^​ Cardiff and Vale University Health Board, Cardiff, Wales, UK; ^4^​ Wales Specialist Virology Centre, Microbiology, Public Health Wales, Cardiff, Wales, UK; ^5^​ Cardiff University School of Biosciences, Cardiff University, Cardiff, Wales, UK

**Keywords:** COVID-19, genomics, recombination, SARS-CoV-2, surveillance

## Abstract

Recombination, the process whereby a segment of genetic material from one genome is inserted into another, producing a new chimeric genome, is an important evolutionary mechanism frequently observed in coronaviruses. The risks posed by recombination include the shuffling of advantageous mutations that may increase transmissibility, severity or vaccine escape. We present a genomic and epidemiological description of a new recombinant lineage of severe acute respiratory syndrome coronavirus 2 (SARS-CoV-2), XR, first identified in Wales. The Pathogen Genomics Unit (Public Health Wales, UK) sequences positive SARS-CoV-2 PCR tests using the ARTIC SARS-CoV-2 sequencing protocol. Recombinants were detected using an in-house pipeline and the epidemiological data analysed in R. Nosocomial cases were defined as those with samples taken after >7 days in hospital. Between February and March 2022, we identified 78 samples with highly similar genomes, comprising a BA.1-like 5' end, a BA.2-like 3' end and a BA.2-like spike protein. This signature is consistent with recombination and was defined as XR by Pangolin (PANGO v1.8). A total of 50 % of cases had a sample collected whilst in hospital and the first three cases were immunocompromised patients. The patient median age was 58 years (range: 4–95 years) and most of the patients were fully vaccinated against SARS-CoV-2 (74 % third dose/booster). Three patients died within 28 days of their sample collection date, one of whom had COVID-19 listed amongst ICD10 (International Classification of Diseases 10) coded causes of death. Our integrated system enabled real-time monitoring of recombinant SARS-CoV-2 for early detection, in order to rapidly risk assess and respond. This work highlights the importance of setting-based surveillance of recombinant SARS-CoV-2, as well as the need to monitor immunocompromised populations through repeat testing and sequencing.

## Data Summary

All sequence data used in this study can be accessed from the GISAID database. Table S1 (available with the online version of this article) contains the sequence accession numbers.

**Impact Statement**

We describe the detection of a new recombinant severe acute respiratory syndrome coronavirus 2 (SARS-CoV-2) lineage encompassing Omicron sublineages BA.1 and BA.2 (termed lineage XR). Recombination events pose a significant risk, as shuffling of advantageous mutations may produce novel phenotypes that can enhance transmissibility, severity or confer vaccine escape. This article describes cases of lineage XR by underlying test results, demographics, vaccination status, travel and severe outcome status [hospitalization, admission to intensive care unit (ICU) and death]. Our retrospective epidemiological investigation revealed that 50 % of XR cases had a sample collected whilst in hospital and that the first 3 cases of the recombinant lineage were in patients who were immunosuppressed. This finding adds to the growing evidence that recombination events are more likely to take place in hospital settings, particularly in wards where immunosuppressed inpatients reside long term. It is important to raise awareness of the recombination potential in this population, and we recommend regular testing and genomic surveillance of SARS-CoV-2 in hospitalized, immunosuppressed patients with COVID-19. Wales has devolved healthcare including surveillance and health protection, with a land border with the larger English nation, and a population of 3.1 million. The methods and findings here are applicable to other countries with independent genomic surveillance for SARS-CoV-2.

## Introduction

The causative agent of COVID-19, severe acute respiratory syndrome coronavirus 2 (SARS-CoV-2), has evolved into phylogenetically distinct lineages since it first emerged in 2020 [1]. New lineages are typically characterized by nucleotide substitutions, insertions or deletions, but genetic recombination has also been reported [2]. Recombination, an important evolutionary mechanism frequently observed in coronaviruses, is the process whereby a segment of genetic material from one genome is inserted into another, producing a new chimeric genome [3, 4].

Recombination events pose a significant risk, as shuffling of advantageous mutations may produce novel phenotypes that can enhance transmissibility, severity or confer vaccine escape. These present a particular concern when occurring between highly prevalent lineages, often designated a ‘variant of concern’ (VOC) [5, 6], which have each caused a specific epidemic [7]. The ability to detect recombination events among SARS-CoV-2 is difficult due to the low degree of differences between isolates [8]. It is important to monitor the incidence and drivers of SARS-CoV-2 recombinants, especially at a time when countries are evaluating their testing strategies.

The Omicron (B.1.1.529) VOC was first reported in Wales on 3rd December 2021 and became the dominant variant by 29th December 2021. Omicron has since divided into multiple sublineages (e.g. BA.1, BA.2, BA.3, BA.4 and BA.5), four of which are also designated as VOCs by the UKHSA (UK Health Security Agency). In this study, we present a genomic and epidemiological description of a new recombinant lineage, XR, first identified in Wales. This lineage contains mutations from Omicron sublineages BA.1 and BA.2, and was first identified in three immunocompromised individuals at risk of severe COVID-19 disease. The lineage was flagged by the Public Health Wales Pathogen Genomics Unit and a retrospective epidemiological investigation was undertaken by the Communicable Disease Surveillance Centre (Public Health Wales) to identify any risk factors for these cases.

## Methods

SARS-CoV-2 real-time PCR testing was conducted in line with local guidelines. The Public Health Wales Pathogen Genomics Unit sequences all samples with a cycle threshold ≤30 using the ARTIC nCoV PCR protocol with ARTIC v4.1 primers [9]. Samples sequenced by Public Health Wales were prepared using the Nextera XT DNA library preparation kit and sequenced on Illumina NextSeq 550 systems. Seven additional samples that were associated with Welsh patients were not sequenced by Public Health Wales and were sequenced by the Wellcome Sanger Institute. These samples were sequenced on an Illumina NovaSeq 6000 using the ARTIC nCoV PCR protocol with ARTIC v4.1 primers [9]. The Public Health Wales Pathogen Genomics Unit was a key site for the COG-UK (COVID-19 Genomics UK Consortium) sequencing programme, and sequences were uploaded to MRC CLIMB [10, 11] to allow UK-wide collation, management and processing. Sequences were subsequently analysed using an automated phylogenetics pipeline that assigns cases to a PANGO lineage [11] and a putative ‘UK transmission group’ using ancestral state reconstruction [1, 12]. Recombinants were confirmed using an in-house pipeline based on the 3SEQ Recombination Detection Algorithm version 1.8 [13]. The 3SEQ analysis was carried using the –triplet variable, comparing against BA.1 and BA.2 reference samples (Table S1), setting these reference samples as parental lineages.

The schematic representation of the XR lineage was generated in Python 3.6.9 using the DNA Features Viewer package v3.1.2 [14]. The phylogenetic tree was generated using iq-tree v2.0.6 (parameters: -m HKY -czb -blmin 0.0000000001 -nt 1 -fast) [15] and plotted using ggtree v2.4.1 in R v4.1.3 [16]. The epidemiological curve and timeline chart were plotted using the ggplot2 package v3.4.0 in R v4.1.3 [17]. The ‘snipit’ plot was generated with snipit using the recombi-mode option (no release version available, commit 56f71ea: https://github.com/aineniamh/snipit) [18]. Descriptive analysis was performed using R v4.1.3.

The Welsh Immunisation System was used to ascertain the patient vaccination status for confirmed cases. An individual was considered to be ‘vaccinated’ against SARS-CoV-2 (with two doses) if they had had two doses of vaccine 14 days prior to their sample date. icnet was used to identify patient admissions to hospital and determine likely acquisition status. icnet is a hospital infection prevention case management and reporting system used across Wales by infection prevention and control (IPC) teams and for systematic surveillance by Public Health Wales. An admission was classified as an individual with a positive PCR result for COVID-19, sequenced as XR, who was admitted to hospital on or 1 day before the day of their first positive test, or in the 28 days following a positive test. Likewise, admission to intensive care unit (ICU) status was based on an individual being identified in icnet as having been admitted to ICU on or 1 day before the day of their first positive test, or in the 28 days following a positive test. Nosocomial cases were defined as ‘definite healthcare-acquired infection’ (HAI) if a positive test was identified ≥15 days post-admission, ‘probable HCAI’ if 8–14 days post-admission, ‘indeterminate’ if 3–7 days post-admission and ‘community-acquired infection (CAI)’ if <48 h post-admission. These are in line with European and nationally agreed surveillance definitions for COVID-19 [19].

Vital status was determined by linking to the Public Health Wales Rapid Mortality Surveillance data. This surveillance is based on clinician reported deaths in confirmed cases of COVID-19 from hospitals or care homes, where the clinician suspects COVID-19 is a causative or contributory factor in the death. We also carried out confirmation of deaths against ICD10 (International Classification of Diseases 10) coded death certificates from the Office of National Statistics (ONS) in order to identify additional deaths that may have occurred outside these settings.

## Results

### Phylogenetic investigation

**Fig. 1.**
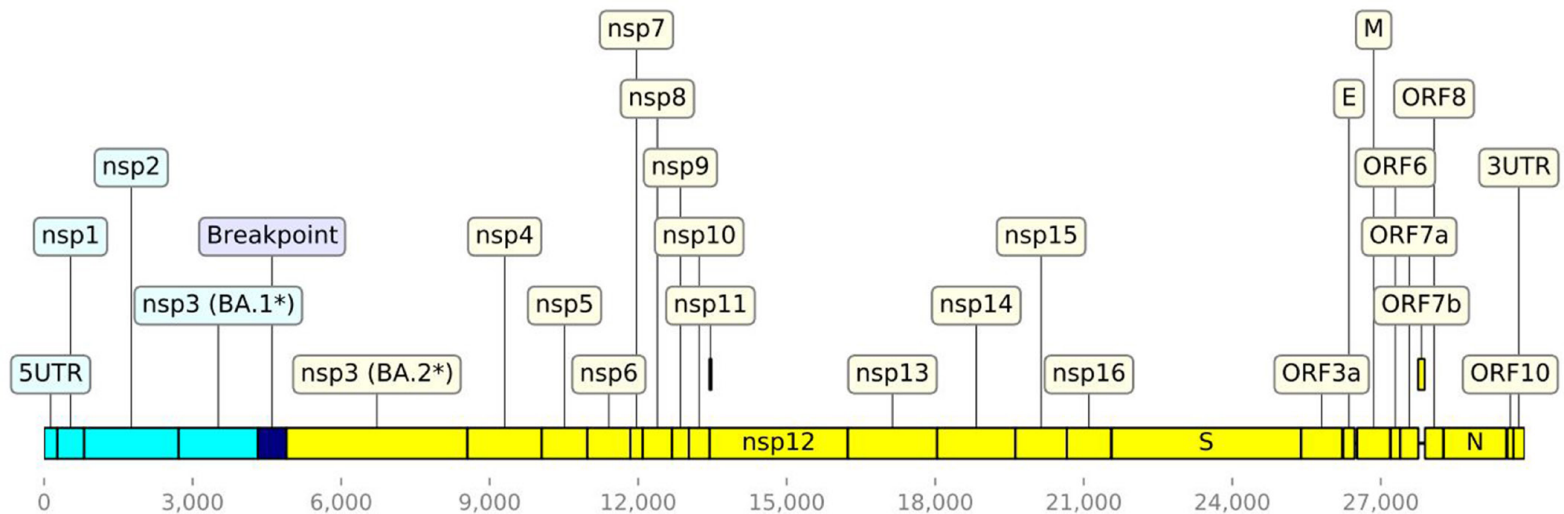
Schematic representation of the SARS-CoV-2 XR recombinant lineage.

Between 13 February and 24 March 2022, 78 samples with highly similar genomes were identified. Two samples were identified from individuals living outside Wales, which were not included in the epidemiological investigation. In total, there were 18 unique sequences identified across the 78 samples, excluding ambiguous base calls. Nine of these were unique to one sample. The largest group of identical sequences comprised 21 samples. Median group size for groups where *n*>1 was 5. The 78 recombinant genomes comprised a BA.1-like 5' end, a BA.2-like 3' end and a BA.2-like spike protein (Fig. 1). This signature is consistent with recombination and was defined as XR by Pangolin [20]. The breakpoint was estimated at 4321–4892 bp, within the Orf1ab nsp3 protein. Fig. S1 depicts the SNPs found in each query sequence compared to the reference sequence, as well as compared to an example BA.1 and BA.2 sequence, each chosen as representative of their lineage based on their high sequencing quality (Fig. S1).

**Fig. 2.**
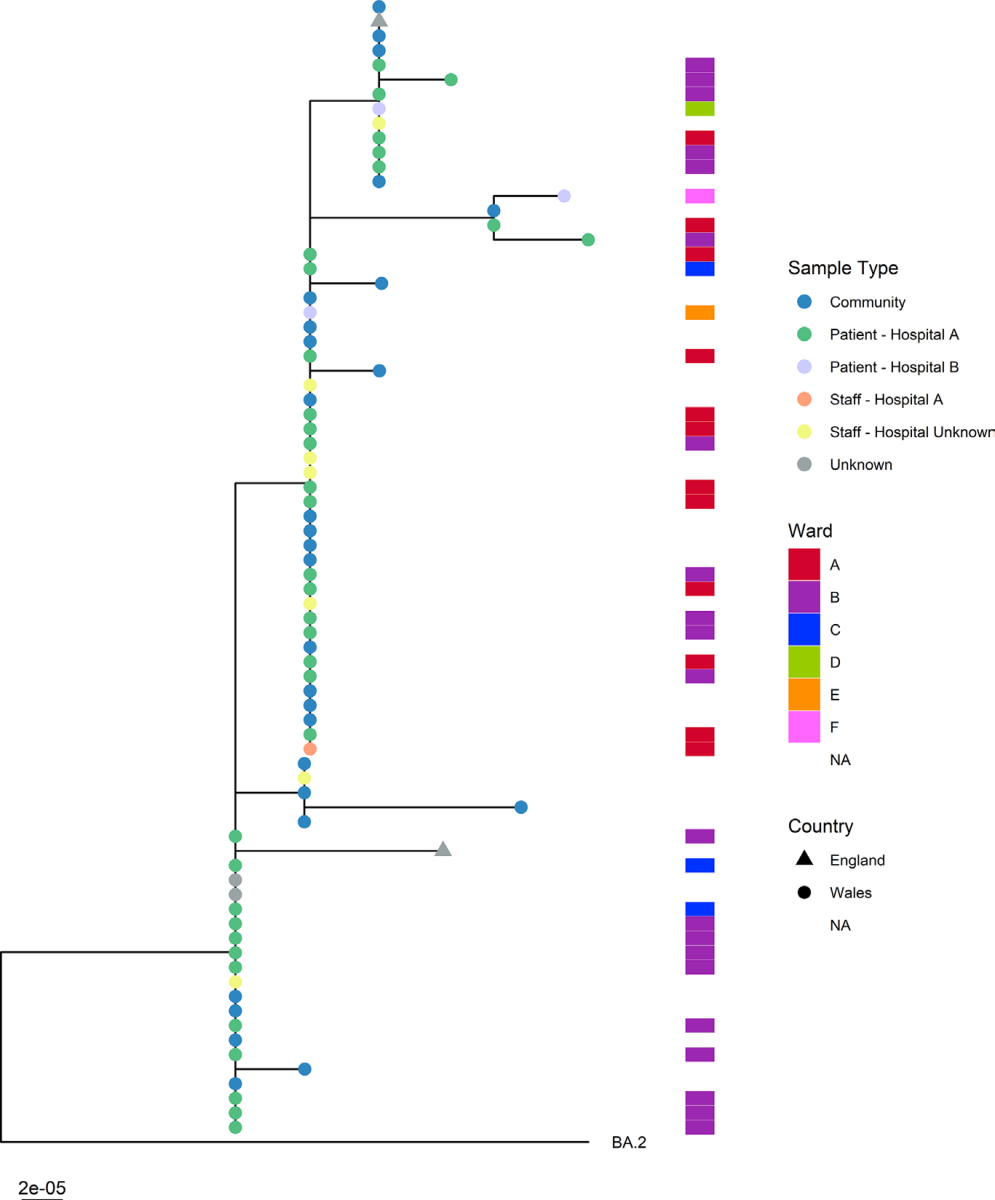
Phylogenetic tree of all SARS-CoV-2 lineage XR recombinant genomes using a representative BA.2 sequence as the reference; node shape indicates country of residence, node colour depicts sample type, left bars indicates wards where applicable. NA indicates data type is not relevant to the sample in question. UK, 13 February – 24 March 2022, data as at 9 August 2022 (*n*=78).

The mean sequencing depth of variant positions across samples compared to the Wuhan-Hu-1 isolate was 2295 reads, and the mean prevalence of the majority nucleotide at these positions was 97.8 %. The mean sequencing depth across the 78 XR samples in positions where either the BA.1 or BA.2 representative genomes harboured private mutations was 2340 reads, and the prevalence of the majority nucleotide at these positions was 99.3 %. Of the 78 XR samples, 15 had an alternative nucleotide frequency of <75 % at the BA.2 private mutation position 2832. All 15 sequences, however, shared the same majority nucleotide with BA.2, at frequencies of >75 %, at each of the five BA.1 private mutation positions in the proposed BA.2 region of the genome, before the estimated breakpoint. This rules out the possibility of both BA.1 and BA.2 lineages being concurrently present in the sample as a result of either co-infection or sample contamination. In addition, all 15 samples shared high sequence concordance with the remaining 56 XR samples, differing from the closest samples by 1–2 SNPs (Fig. 2).

### Epidemiological investigation

A retrospective epidemiological investigation was undertaken to establish any epidemiological links between the 76 cases of patients residing in Wales. Initial findings revealed that cases were predominantly identified in one health board; 65 patients reported no overseas travel (travel data not available for 11 patients). Overall, the patient median age was 58 years (range: 4–95 years) and the majority were female (64%). The majority of patients were fully vaccinated against SARS-CoV-2 (74 % third dose/booster) with the remainder having a first dose only (*n*=4), having a second dose only (*n*=10), being unvaccinated (*n*=5) or of unknown vaccination status (*n*=1). Overall, three patients with the XR lineage died within 28 days of their sample collection date, one of whom had COVID-19/respiratory infection listed amongst ICD10 coded causes of death. Ethnicity information was poorly completed; however, 24 individuals reported ‘English, Welsh, Scottish, Northern Irish or British’, 3 individuals reported ‘Indian’ and 1 individual reported ‘Chinese’ ethnicity.

**Fig. 3.**
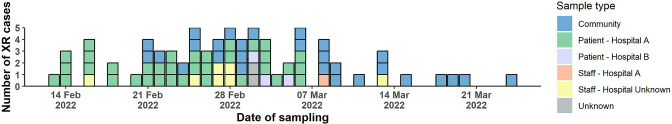
Sample date for SARS-CoV-2 XR cases by sample type. Wales, 13 February – 24 March 2022, data as at 9 August 2022 (*n*=76).

In total, 50 % of patient cases had a sample collected whilst an inpatient in hospital; 35 cases in hospital A and 3 cases in hospital B (Fig. 3). Within hospital A, 31 % of patient cases were associated with ward A (*n*=11), 60 % with ward B (*n*=21) and the remaining 9 % with ward C (*n*=3) (Fig. 2). In hospital A, 19 cases were defined as definite HCAIs and 10 as probable HCAIs. In hospital B, one case was identified in each of ward D, ward E and ward F, and all three cases were defined as definite HCAIs (Fig. 2). The remaining samples were classified as ‘community’ (*n*=30) and ‘staff’ (*n*=8). Within the staff samples, one sample was associated with hospital A, ward A. It was not possible to confirm the hospital or ward locations of the remaining seven staff samples (Fig. 3).

An outbreak was declared on ward B of hospital A on the 14 February 2022 that lasted 25 days and involved 27 patients and 7 members of staff (sequencing coverage: 62%). An outbreak was declared in ward A of hospital A on the 20 February 2022 lasting 19 days, which involved 25 patients and 7 members of staff (sequencing coverage: 34%). Ward A and ward B are adjacent to each other in the hospital and during periods of increased pressures staff may be shared between wards, suggestive of a single outbreak of the XR lineage.

**Fig. 4.**
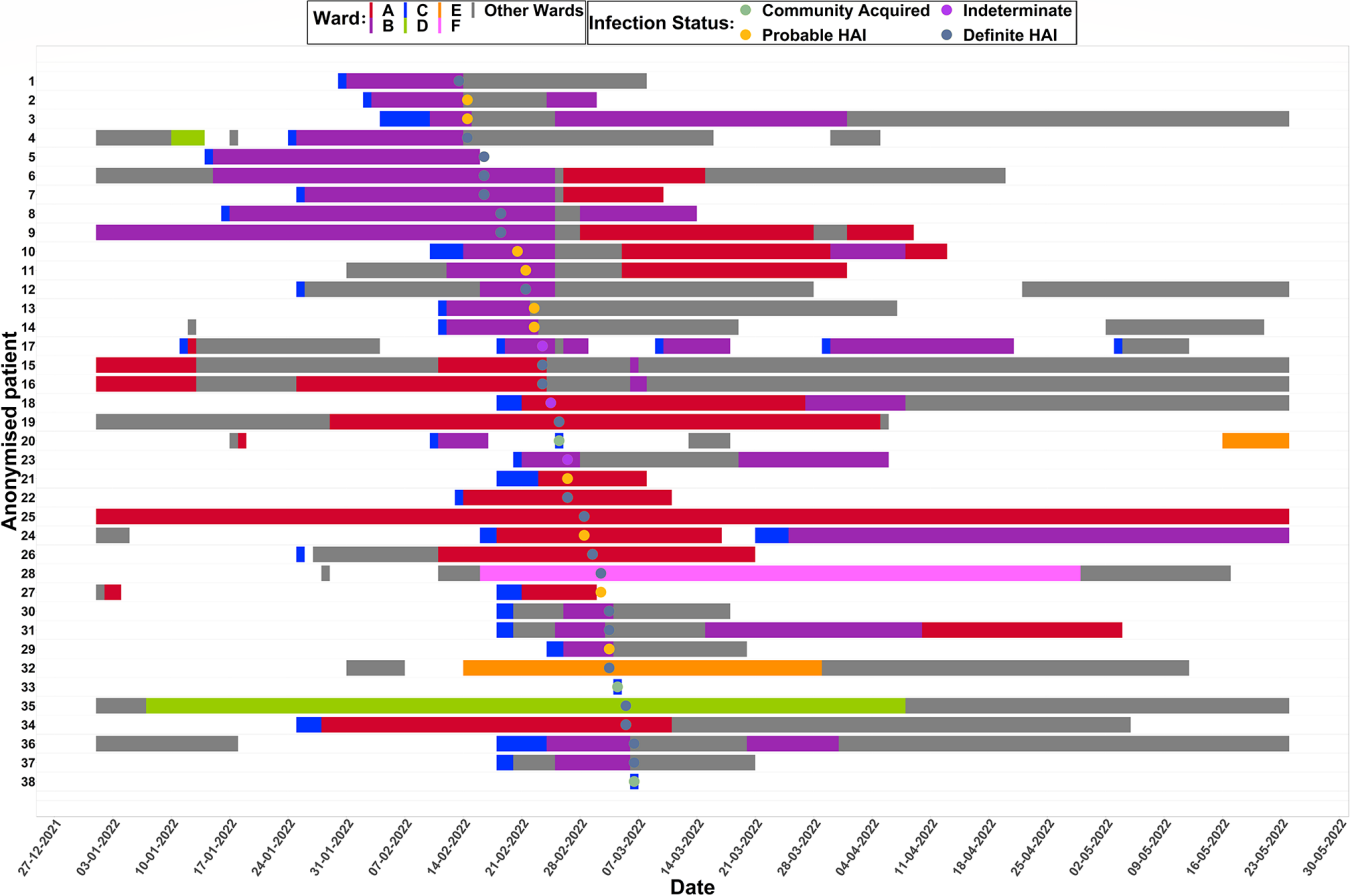
Timeline chart depicting patient movements across wards in hospital A and hospital B for patients with lineage XR, Wales, 27 December 2021 – 30 May 2022. Dots indicate date of COVID-19 positive test and their colour indicates HAI category (green indicates community-acquired infection, blue indicates indeterminate, yellow indicates probable HAI, pink indicates definite HAI). Bar colour indicates ward (note that the grey bar, ‘other wards’, includes wards within hospital A and hospital B). Data as at 9 August 2022 (*n*=38).

No links were identified between patients in hospital A and hospital B. The declared outbreaks in hospital A indicate that the recombinant lineage may have been more widespread than initially estimated using the genomic surveillance system alone. Furthermore, Fig. 4 shows that the first 14 cases of sequenced XR were either definite HAI or probable HAI, indicating that the recombinant lineage likely originated in hospital A and transmitted to hospital B through an unsampled case within the community or healthcare system. The initial case of XR, identified on 13 February 2022, was in an immunocompromised patient. Three cases were subsequently identified on 14 February 2022, two of which were also immunocompromised patients. Interrogation of the sequencing data revealed that the XR lineage was not identified elsewhere in Wales, indicative of a point source outbreak as opposed to multiple introductions of a new lineage.

## Discussion

This work adds to the growing evidence demonstrating the recombination potential of SARS-CoV-2 [2, 21–24]. Recombination can occur when an individual is coinfected by two genetically distinct viruses. This is more likely to occur whilst there is a high prevalence of SARS-CoV-2 in the population and there is co-circulation of multiple variants [2]. Estimates for the week ending 12 February 2022 (the week preceding the first identified case of XR) reveal that 1 in 25 people (4.13 % of the population) had tested positive for COVID-19 in Wales [25]. Moreover, the Wales whole genome sequencing (WGS) surveillance data indicate that BA.2 became the dominant variant (taking over from BA.1) in the week ending 26 February 2022. This high prevalence, in combination with the co-circulation of the Omicron sublineages, BA.1 and BA.2, allowed for optimal conditions for a new recombinant lineage to emerge.

This study revealed that the recombination event likely occurred in a hospital setting where inpatients were residing long term. Moreover, the initial cases of the XR lineage were identified in immunocompromised patients with the first case being a definite HAI. We are also aware that during this time period there were a number of outbreaks of BA.1 and BA.2 within the hospital. As restrictions had been lifted at this time, visitors were permitted to visit the hospital, which may explain how the patient came to be co-infected with both BA.1 and BA.2. This observation is in line with current hypotheses that recombination events are more likely to take place following sustained exposure of susceptible, immunosuppressed hosts during periods of widespread community circulation of variants [23, 26]. Due to the very close temporal timing and that all three immunocompromised patients resided on the same ward, it is likely that this lineage arose in one of these patients and onward transmission occurred in this group. We recommend targeted sampling strategies for genomic surveillance aimed at inpatient and immunocompromised patients in order to monitor the genomic data for new recombinant lineages and initiate investigations in a timely way [27, 28]. We also recommend that booster vaccination doses for SARS-CoV-2 are offered to individuals living with immunosuppressing conditions, in line with guidance.

There is no evidence that the recombinant XR lineage confers increased transmissibility or virulence. This may be due to the emergence of this recombinant during a period of high BA.2 circulation, or because the genome structure of this recombinant is primarily BA.2-like, with a small BA.1-like fragment at the 5' end. Furthermore, there is no evidence in this study that lineage XR resulted in more severe outcomes for those infected. Although it should be noted that comparison of severity is impacted by a number of factors, including comorbidities and vaccination status. Whilst most recombination events produce unviable genomes, they have the potential to introduce dramatic changes to the genome at a much faster rate than through standard mutation alone.

The identification of recombinant SARS-CoV-2 lineages relies on high genomic surveillance capabilities. The cumulative WGS coverage of Welsh SARS-CoV-2 cases up to the 24 March 2022 over the course of the pandemic was 32.5%, and over the course of the 6 weeks from the 13 February to the 24 March 2022, 69.0% of all Welsh cases were sequenced. Equivalent data from England indicate that from 13 February to the 24 March 2022, 38.6% of all English cases were sequenced [29]. As a result, although we are confident that XR was likely not circulating widely within Wales, we may not be as confident in this assumption for England due to the lower sequencing coverage. Generally, the sequencing coverage demonstrated in Wales is not available globally and countries with limited established sequencing capacity are less likely to detect recombination events. This would suggest that recombinant variants of SARS-CoV-2 are more common than is reported worldwide. Furthermore, the limited availability of publicly available epidemiological, demographical and clinical information on sequenced lineages limits the analyses on outcomes, disease severity and vaccine efficacy [30]. Despite the XR lineage not becoming dominant, SARS-CoV-2 recombination events should continue to be monitored in order to detect new lineages that may alter the pathogenicity of the virus. This is particularly relevant for the design of vaccines, drugs and diagnostic tools.

## Limitations

The identification of recombinant lineages is limited by the small number of phylogenetically informative sites. The use of strict data quality criteria to reduce contamination events and sequencing artefacts may, by design, omit potential recombination events due to the presence of mixed viral populations. As a result, this study may underestimate the number of XR recombinant samples present in Wales.

The results are also limited by the availability of sequencing data. On the 28 March 2022, community PCR testing was stood down following a change in testing policy. This resulted in a vast reduction of sequencing results, impacting the ability to detect recombinant lineages. As the lineage was likely transmitted between hospital A and hospital B through an unsampled case, it is likely that this study underrepresents the true number of XR cases within Wales. This issue is compounded by the current testing strategy whereby the majority of sequenced samples are from hospital inpatients with 50.3 % of hospitalized cases in Wales successfully sequenced (28 March – 9 August 2022).

## Conclusion

Our study demonstrates the utility of WGS for the identification of new recombinant lineages. Our integrated system enabled real-time monitoring of recombinant SARS-CoV-2 for early detection in order to rapidly risk assess and respond. As recombination requires coinfection of patients, healthcare settings provide a key area where recombinants may arise. This study demonstrates that a recombinant lineage likely arose in a patient who was immunocompromised. We recommend regular testing and genomic surveillance of SARS-CoV-2 in hospitalized, immunosuppressed patients with COVID-19. We also recommend that individuals living with immunosuppressing conditions are offered booster vaccination doses for SARS-CoV-2, in line with guidance, in order to prevent, where possible, transmission of COVID-19, particularly in inpatient settings.

## Supplementary Data

Supplementary material 1

Supplementary material 2
