# Supplementary material for: Novel recombinant SARS-CoV-2 lineage detected through genomic surveillance in Wales, UK
Source: Microb Genom. 2023 Apr 13;9(4):mgen000984. doi: 10.1099/mgen.0.000984 (PMC10210960; doi:10.1099/mgen.0.000984)
Supplement: Supplementary material 1 [file mgen-9-984-s001.pdf]

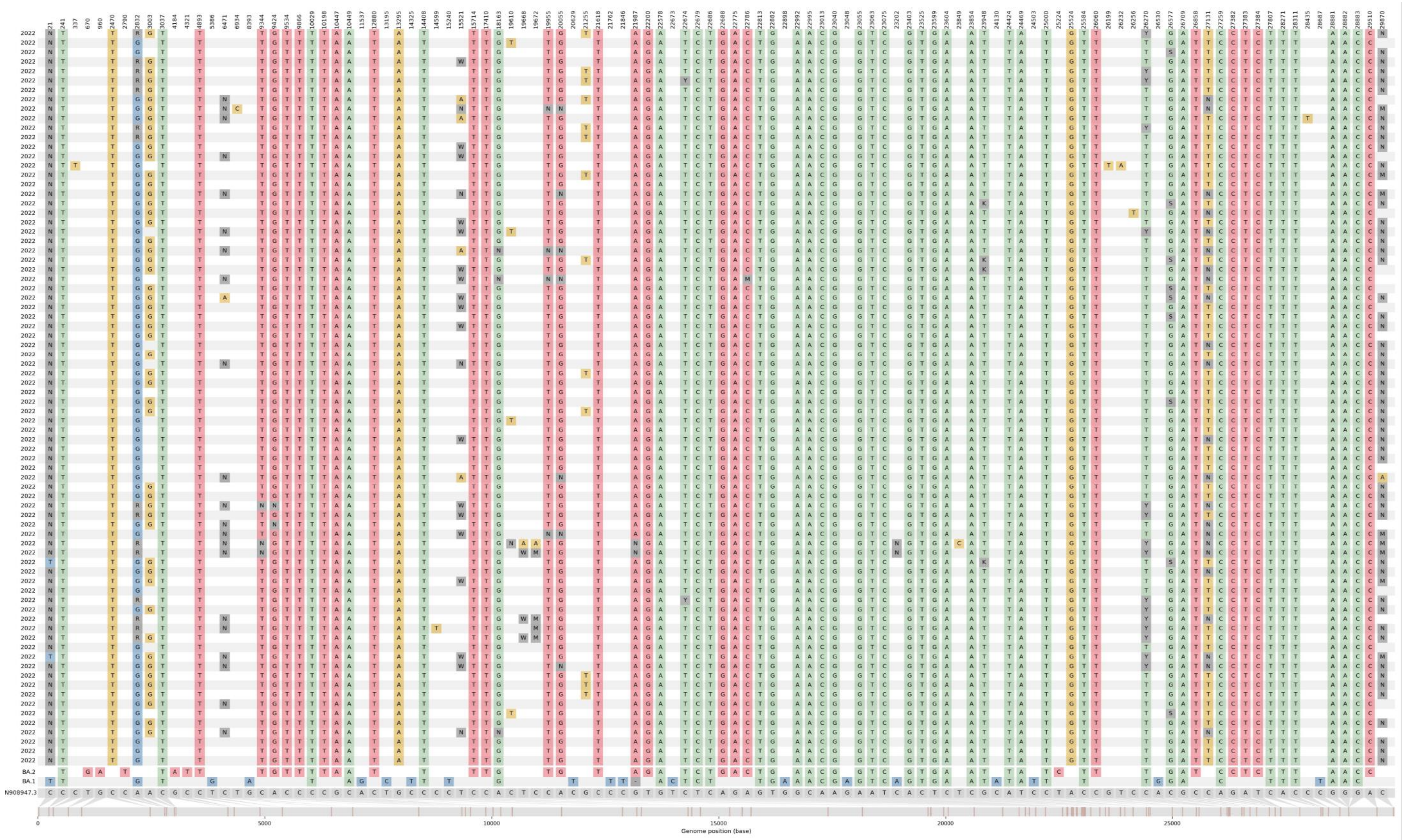

Supplementary Figure 1. 'snipit' plot of XR samples showing the single nucleotide polymorphisms (SNPs) found in each query XR sequence compared to the reference sequence as well as an example BA.1 and BA.2 sequence
